# Use of filter paper stored dried blood for measurement of triglycerides

**DOI:** 10.1186/1476-511X-5-20

**Published:** 2006-07-14

**Authors:** Rizwana Quraishi, Ramakrishnan Lakshmy, Dorairaj Prabhakaran, Ashok Kumar Mukhopadhyay, Bansilal Jailkhani

**Affiliations:** 1 Laboratory Medicine department, All India Institute of Medical Sciences (AIIMS), New Delhi, India; 2 Department of Cardiac Biochemistry, CNC, AIIMS, New Delhi, India; 3 Cardiology department, CNC, AIIMS, New Delhi, India

## Abstract

Adaptation of assays on dried blood has advantages of ease of collection, transportation, minimal invasiveness and requirement of small volume. A method for extraction and estimation of triglyceride from blood spots dried on filter paper (Whatman no. 3) has been developed. A single dried blood spot containing 10 μL blood was used. Triglyceride was efficiently extracted in methanol from blood dried on filter paper by incubation at 37°C for two hours with gentle shaking. For the estimation, a commercially available enzymatic method was used. Blood spot assays showed mean intra and inter assay coefficient of variance of 6.0% and 7.4% respectively. A comparison of paired whole blood spots and plasma samples (n = 75, day 0) gave an intraclass correlation of 0.96. The recovery was 99.6%. The dried blood triglyceride concentrations were stable for one month when the filter discs were stored at room temperature (16–28°C). Storage of filters at 4°C extended the stability and triglycerides could be quantatively recovered after 3 months of storage.

## Findings

Serum triglyceride (TG) is an important risk factor for heart diseases [1-3]. Large scale epidemiological evaluation of TG poses several challenges in developing countries. Storage and transportation of the samples from field is a limiting factor, especially in areas lacking modern laboratory facilities. A method that eliminates the need to store and ship samples at low temperatures would therefore be desirable. Assays using whole blood dried on filter paper may provide a viable alternative. Several community based studies have shown this to be convenient and reliable for evaluation of a number of analytes [4-8]. We describe here a method for extraction and measurement of TG from blood samples collected on Whatman no. 3 filter paper (Whatman international Ltd, England.) Seventy five patients visiting the centralized laboratory, Cardio-Neuro Center, All India Institute of Medical Sciences (AIIMS) for lipid investigations were selected at random. Blood was collected by venipuncture into tubes with anticoagulant. Ethical clearance for the conduct of the study was obtained from institutional (AIIMS) ethics committee. Blood spots were prepared by pipetting 10 μL of the blood onto the filter paper kept on a nonabsorbent surface (thermacol) and left at room temperature for drying. The room temperature was 16–28°C for the duration of study. After drying, the filter discs were kept in sealed plastic bags to protect them from dust and moisture, and stored at 4°C or at room temperature (16–28°C) for 3 months.

Plasma was also separated from each blood sample and analysed on the day of collection for comparison. For dried blood lipid measurement, one disc corresponding to 10 μl of blood was cut and put in a stoppered test tube and 100 μl of methanol (Analytical grade, Qualigens, Glaxo limited, India) was added. The tubes were incubated at 37°C for 2 hours, with shaking at 100 rotations per minutes in an environ shaker (Lab Line Inc. ILL, USA). For estimating triglycerides in the eluate, 50 μL of the extract was added to 1 ml of the commercially available enzymatic reagent kit (Lot no. 773TR, Randox) [9]. The reaction mixture was stirred and incubated at 37°C for 15 min, and measured at 500 nm (OD_500_) on a spectrophotometer (Spectronic instruments Inc. New York, USA) using whole blood zero standard as blank. Plasma TG was determined with the same kit. To minimize matrix differences and maximize comparability between calibrators and test samples, dried blood spot standards and controls were prepared by mixing washed red blood cells [10] with the TG standard provided with the enzymatic kit (Lot no.636TR, Randox) and quality controls level I to III (Lot no.1392, 1462 & 1387 CH, Randox). The lipid standard was serially diluted in normal saline (0.8 g NaCl in 100 ml, of deionized H_2_O) and washed erythrocytes were mixed in proportions 50:50 (v/v) to get whole blood calibrators with triglyceride concentrations 1.15, 0.573, 0.287, 0.144 and 0.00 mmol/L. The blood based quality controls were similarly prepared by adding washed erythrocytes in 1:1 dilutions. Ten micro liters of each whole blood standard, control and blank were spotted on filter paper and allowed to dry at room temperature for 1 hour.

**Figure 1:**
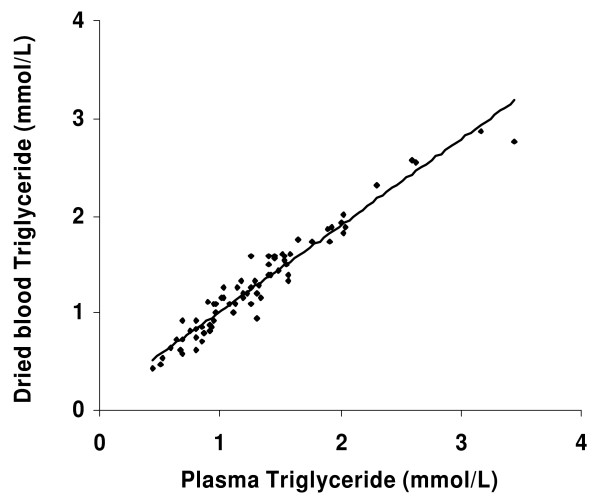
**Relationship between dried blood and plasma triglyceride concentrations in 75 paired samples**. Regression curve for triglyceride measurement in fresh plasma and blood dried on filter paper. Triglyceride values (n = 75) obtained in dried blood were correlated with values obtained in fresh plasma. Equation for the line is y = 0.88x + 0.13 (r = 0.97, P < 0.001). The intraclass correlation of the paired samples (ICC) is 0.96. Values represent the mean of duplicate measurement.

Intra and inter assay coefficient of variance of the modified method for dried blood was 6.05 % and 7.44 % respectively. The relationship between dried blood and plasma analysed on the day of collection was linear and highly correlated with r value of 0.97 and Intraclass correlation (ICC) value of 0.96 (Fig. 1). The total triglyceride values in the 75 plasma samples ranged from 0.44 mmol/L to 3.45 mmol/L. The TG mean value on the day of sample collection was 1.318 ± 0.58 and 1.297 ± 0.53 mmol/L in plasma and dried blood respectively. Mean recovery from dried blood was 99.6 %. There were 61 samples with TG values <1.725 mmol/L and 14 samples had values >1.725 mmol/L.

To assess stability, the triglyceride concentrations were analyzed at various time intervals in the 75 dried blood samples by the method described above. The triglyceride concentration in dried blood samples stored for different time periods were compared with plasma values on the day of collection using within subject analysis of variance (ANOVA). Triglyceride concentration remained stable for 30 days at 16–28°C (F = 1.228, P = 0.296) and for at least 90 days at 4°C (F = 0.496, P = 0.483). At 60 day the TG level was 1.536 ± 0.54 as compared 1.318 ± 0.58 mmol/L on day 0 (F = 37.569, P < 0.000).

Total hematocrit (hct) values in the 75 samples ranged from 21% to 49%. The samples were categorized into 3 groups (I- hct < 30%, II- between 30–40% and III- 40–50%) to assess the effect of hematocrit. The mean TG values between plasma and dried blood samples in the three hct groups were not different statistically. Paired t-test 'p' values with corresponding plasma and dried blood TG mean concentrations were; group I- p = 0.43,1.93 ± 1.25 and 1.77 ± 1.00 mmol/L (n = 4), group II- p = 0.93, 1.30 ± 0.54 and 1.30 ± 0.50 mmol/L (n = 46) and group III- p = 0.132, 1.26 ± 0.47 and 1.21 ± 0.46 mmol/L (n = 25) respectively. No effect of hematocrit was noted in this study.

We have earlier studied the stability of dried serum at room temperature for cholesterol and triglyceride measurement and found the analytes to be stable up to 35 days [11]. The widespread use of dried serum in community-based studies has the limitation of a need for field processing and centrifugation to obtain serum. These constraints can be overcome with the use of dried blood. Previous studies of dried blood assays for the measurement of cholesterol from dried blood reported a significant correlation between the serum and the dried blood spots [12]. However, a lower recovery and low upper limit values was reported by Bradbury and Forrest [13].

The stability of lipids in dried blood samples at room temperature up to 30 days would have great applicability in developing countries with considerable rural populations who have limited accessibility to diagnostic labs performing the investigations. Further, the adaptation of lipid assays to dried blood is ideal for pediatric applications and is useful in multicentric studies where the cost and safety of sample transportation to a distant laboratory are limiting factors. We conclude that triglyceride can be easily extracted from blood dried on filter paper. The acceptable agreement between values in dried blood and fresh plasma samples supports the validity of the assay.

## Competing interests

The author(s) declare that they have no competing interests.

## Authors' contributions

RQ was responsible for the accomplishment of the study and analyses of the blood samples, RL participated in the designing, planning, coordination and drafting of the manuscript, DP participated in the design of the study and the statistical analysis, BLJ and AKM have been involved in drafting the manuscript and revising it critically for important intellectual content.
